# Central Role of Ubiquitination in Wheat Response to CWMV Infection

**DOI:** 10.3390/v14081789

**Published:** 2022-08-16

**Authors:** Haichao Hu, Linna Cai, Tianye Zhang, Tingting Liu, Yaoyao Jiang, Hanhong Liu, Qisen Lu, Jian Yang, Jianping Chen

**Affiliations:** 1 College of Plant Protection, Hunan Agricultural University, Changsha 410128, China; 2 State Key Laboratory for Quality and Safety of Agro-Products, Institute of Plant Virology, Ningbo University, Ningbo 315211, China; 3 Junan County Bureau of Agriculture and Country, Linyi 276600, China

**Keywords:** ubiquitination, wheat, CWMV, plant–pathogen, plant defense

## Abstract

Ubiquitination is a major post-translational modification (PTM) involved in almost all eukaryotic biological processes and plays an essential role in plant response to pathogen infection. However, to date, large-scale profiling of the changes in the ubiquitome in response to pathogens, especially viruses, in wheat has not been reported. This study aimed to identify the ubiquitinated proteins involved in *Chinese wheat mosaic virus* (CWMV) infection in wheat using a combination of affinity enrichment and high-resolution liquid chromatography-tandem mass spectroscopy. The potential biological functions of these ubiquitinated proteins were further analyzed using bioinformatics. A total of 2297 lysine ubiquitination sites in 1255 proteins were identified in wheat infected with CWMV, of which 350 lysine ubiquitination sites in 192 proteins were differentially expressed. These ubiquitinated proteins were related to metabolic processes, responses to stress and hormones, plant–pathogen interactions, and ribosome pathways, as assessed via Gene ontology and Kyoto Encyclopedia of Genes and Genomes enrichment analyses. Furthermore, we found that the ubiquitination of Ta14-3-3 and TaHSP90, which are essential components of the innate immune system, was significantly enhanced during CWMV infection, which suggested that ubiquitination modification plays a vital role in the regulatory network of the host response to CWMV infection. In summary, our study puts forward a novel strategy for further probing the molecular mechanisms of CWMV infection. Our findings will inform future research to find better, innovative, and effective solutions to deal with CWMV infection in wheat, which is the most crucial and widely used cereal grain crop.

## 1. Introduction

Plants are sessile organisms that are easily influenced by various environmental factors such as temperature, light, and pathogens [1]. Plants have evolved multiple mechanisms to cope with varying environmental factors and stimuli. Protein post-translational modification (PTM) plays an important role in the regulation of plant growth and development and in response to a variety of environmental stresses [2,3]. To date, more than 660 PTMs have been reported in various studies [1,3]. Reversible PTMs, such as ubiquitination, acetylation, methylation, and phosphorylation, are extensively involved in plant growth and stress responses [4,5,6]. Notably, ubiquitination is one of the most prevalent PTMs that is involved in almost all aspects of eukaryotic biology [1,4,5,6]. In recent decades, studies have extensively reported that ubiquitin, a highly conserved protein composed of 76 amino acids, is labeled with substrate proteins through a series of enzymatic cascades. Three key enzymes, ubiquitin-activating enzyme (E1), ubiquitin-conjugating enzyme (E2), and ubiquitin ligase (E3), catalyze these reactions during the ubiquitination process. Importantly, since E3 can specifically recognize and bind substrate proteins, the specificity of protein ubiquitination is primarily determined by E3 [5,7,8,9]. Substrate proteins labeled with ubiquitin usually have different fates, depending on the length and location of the ubiquitin chain. Most substrate proteins with poly-ubiquitin chains are degraded by the 26S proteasome [8,9]. Additionally, recent studies have shown that some ubiquitin-linked proteins are involved in the regulation of DNA replication, protein synthesis, and immune signaling [4,10,11].

Ubiquitination in eukaryotes is characterized by low site-specific stoichiometry, short lifespan, reversible modification, condition-specific expression, and complex ubiquitin conjugation architectures. All these characteristics make it difficult to fully understand the molecular mechanism underlying the ubiquitination process [12,13]. Despite these challenges, in recent years, great progress has been made in identifying and validating effective analytical methods to probe the complex ubiquitination process in plants. Liquid chromatography-tandem mass spectrometry (LC-MS/MS)-based methods have been widely used for proteomic analysis owing to their high sensitivity and specificity [14,15,16]. Consequently, ubiquitinated proteins can now be identified and quantified rapidly. Indeed, thousands of ubiquitin-linked substrates can be identified in one experiment using the protein/peptide affinity coupled with high-performance MS/MS technology. The application of LC/MS-based techniques has helped in determining the roles of ubiquitin-related proteins in plant growth and stress responses [17,18,19,20]. For example, Zhu et al. identified 1638 lysine ubiquitination sites from 916 unique proteins and systematically revealed the role of lysine ubiquitination in the formation of young rice panicles [17]. Similar studies have identified 544 ubiquitination sites from 352 protein substrates in peach leaves. These ubiquitin-linked substrates modulate a variety of essential cellular and physiological processes, including carbon metabolism, histone assembly, translation, and vesicular trafficking [18]. In addition to the development-related ubiquitin substrates identified in plants, a variety of stress-related ubiquitin proteins have also been identified. A total of 3305 lysine ubiquitination sites from 1562 proteins have been identified in *Saccharina japonica* under heat stress. These proteins regulate the ubiquitin-26S proteasome system, ribosomes, and oxidative phosphorylation [19]. Additionally, many lysine-ubiquitination sites and proteins, which modulate protein transportation, ligand recognition, and redox reactions, have been identified in rice seedlings treated with fungal-derived chitin and bacterial-derived flg22 through ubiquitome analysis [20]. The ubiquitination sites in all these studies were identified to further investigate the regulatory mechanisms of ubiquitination in various plant physiological processes.

Wheat is one of the most important cereal crops worldwide, and the annual wheat yield has a critical impact on food security. Pests and pathogen infections are one of the dominant factors responsible for reduced crop yield and quality [21,22]. *Chinese wheat mosaic virus* (CWMV) is one of the main pathogens that causes wheat mosaic disease [22]. CWMV is a member of the *Furovirus* genus that leads to typical mosaic symptoms in infected wheat plants [23,24]. CWMV is maintained in nature through transmission by *Polymyxa graminis*, an obligate parasite in gramineous roots [22,23,24,25]. Previous studies indicate that the CWMV genome is composed of two single-stranded positive-sense RNAs (RNA1 and RNA2). RNA1 consists of 7147 nucleotides and encodes three proteins required for viral replication and movement. RNA2 contains 3564 nucleotides encoding four distinctive proteins, including a major coat protein (CP, 19 kDa) and two minor CP-related proteins (CP-RT, 84 kDa; N-CP, 39 kDa) produced by occasional read-through of the UGA termination codon or translation initiation from a non-standard start codon (CUG), respectively [24,25,26,27,28]. RNA2 also contains a cysteine-rich protein (CRP, 19 kDa), which is hypothesized to act as an RNA silencing suppressor, and movement protein (MP, 37 kDa) [24,25,26,27,28]. In addition, we previously constructed a full-length cDNA clone of CWMV that can infect wheat and *Nicotiana benthamiana* [29].

Growing evidence suggests that the ubiquitin-proteasome system (UPS) plays a key role in pathogen-triggered plant immunity. However, only a few studies have demonstrated the antiviral function of plant UPS against the positive-strand RNA viruses [30,31]. To date, no large-scale ubiquitome profiling has been reported in wheat after CWMV infection. In this study, we systematically investigated potential changes in the expression of ubiquitinated proteins in wheat after CWMV infection via a label-free quantitative strategy involving antibody-based affinity enrichment and high-resolution LC-MS/MS. Our findings will strengthen future research on strategies to deal with CWMV infection in wheat.

## 2. Materials and Methods

### 2.1. Plant Materials and Sample Preparation

In this study, we used *Nicotiana benthamiana* and the wheat (*Triticum aestivum*) cultivar Yangmai158, which is highly susceptible to CWMV, as the research objects. Wheat and *N. benthamiana* seedlings were grown in a greenhouse at 17 °C, with 16 h light/8 h dark, light intensity at 200 μmol m^−2^s^−1^, and 70% relative humidity. Two-leaf stage wheat seedlings were inoculated with CWMV using in vitro transcripts. The inoculation method was described in a previous study, and mock-inoculated plants were used as controls [29]. All samples from the treatment and control groups were collected seven days after CWMV infection and stored at −80 °C for further study. For all treatments and controls, three independent biological replicates were used for each sample.

### 2.2. Protein Extraction and Trypsin Digestion

Approximately 1 g of the sample was ground into a powder using liquid nitrogen, and then four volumes of lysis buffer (8 M urea, 10 mM dithiothreitol, 1% Triton-100, 3 μM trichostatin A (TSA), 50 mM nicotinamide for acetylation (NAM), and 1% protease inhibitor cocktail) were added to the samples. Subsequently, the lysates were sonicated thrice on ice using a high-intensity ultrasonic processor (Scientz, Ningbo, China). Next, debris was removed via centrifugation at 20,000× *g* and 4 °C for 20 min. Following this, total protein was precipitated using cold 20% trichloroacetic acid (TCA) for 2 h at −20 °C. The supernatant was discarded after centrifugation at 12,000× *g* and 4 °C for 10 min, and the precipitate was washed thrice with cold acetone. Finally, the precipitate was resuspended in a buffer (8 M urea, 100 mM NH_4_CO_3_, pH 8.0), and protein concentration was determined using a BCA kit (Abcam, Cambridge, MA, USA) according to the manufacturer’s instructions.

Approximately 100 mg of protein was treated with 5 mM dithiothreitol (DTT) at 56 °C for 30 min and alkylated with 11 mM iodoacetamide (IAA) for 15 min at 25 °C in the dark. The protein samples were then diluted to reduce the quantity of urea under 2 M using 100 mM triethylammonium bicarbonate (TEAB). Finally, the protein samples were first digested overnight at trypsin: protein mass ratio of 1:50 and then for 4 h at a 1:100 trypsin/protein mass ratio.

### 2.3. Affinity Enrichment of the Ubiquitinated Peptides

To enrich the modified peptides, the tryptic peptide samples were fractionated via high-pH reverse-phase high-performance liquid chromatography (HPLC) using a Dionex Ultimate 3000 instrument (Thermo Fisher Scientific, Rockford, IL, USA, and a Thermo Betasil C18 column (5 μM particles, 10 mm ID, and 250 mm length; Rockford, IL, USA). Subsequently, the peptides were separated into 60 fractions using a gradient of 8–32% acetonitrile (pH 9.0) for 60 min. The peptides were then combined into four fractions and dried via vacuum freezing.

As ubiquitinated proteins contain a unique di-Gly remnant (K-ε-GG), a high-quality anti-K-ε-GG antibody can be used to enrich the ubiquitinated proteins. The lyophilized peptides were dissolved in an NETN buffer (100 mM NaCl, 1 mM EDTA, 0.5% NP-40, and 50 mM Tris-HCl, pH 8.0). The dissolved peptides were incubated with pre-washed anti-K-ε-GG antibody beads (Lot number 001, PTM Bio, Hangzhou, China) and gently shaken overnight at 4 °C; the beads were then washed four times with NETN buffer and twice with deionized water. The bound peptides were eluted thrice with 0.1% trifluoroacetic acid, and the products were freeze-dried. For LC-MS analysis, the obtained peptides were desalted using C18 Zip Tips (Millipore, Darmstadt, Germany) according to the manufacturer’s instructions.

### 2.4. LC-MS/MS Analysis

To dissolve the lyophilized peptide samples, 0.1% formic acid (solvent A) was used, and the samples were then directly loaded onto a reversed-phase analytical column (15 cm in length and 75 μm inside diameter (*i*.*d*.)). The peptide mixture was separated using a linear gradient of solvent B (0.1% formic acid in 98% acetonitrile) at a constant flow rate of 400 nL/min. In this study, we performed LC-MS using the Q Exactive ^TM^ Plus Orbitrap Mass Spectrometer (Thermo Fisher Scientific, Rockford, IL, USA) and the Proxeon Biosystems Easy nLC^TM^ system (Thermo Fisher Scientific). Parameters were set as follows: electrospray voltage, 2.0 kV; automatic gain control (AGC), 5E4; survey scans were acquired at a resolution of 70,000; resolution for HCD spectra, 17,500; isolation width, 2 *m*/*z*.

### 2.5. Database Search and Bioinformatic Analysis

The resulting LC-MS data were processed by the MaxQuant search engine (v. 1.5.2.8) [32]. Tandem mass spectra were searched against the *Triticum aestivum* database (http://plants.ensembl.org/Triticum_aestivum/Tools/Blast, accessed on 8 May 2021) concatenated with a reverse-decoy database. Trypsin/P is a specific enzyme that allows four missed cleavages. During the database search, the mass tolerance for the precursor ions was set at 20 and 5 ppm in the first and main searches, respectively. Additionally, the mass tolerance for fragment ions was set as 0.02 Da [33]. In addition, the false discovery rate (FDR) was adjusted to less than 1%, and the minimum score for the modified peptides was set to >40. The PCA, RSD, and Pearson’s correlation coefficient were used to determine whether the agreement between biological duplications among the treatment and control groups was statistically consistent.

Bioinformatic analysis of the ubiquitinome data was performed according to previously described methods [16,17,18,19,20,21]. The Uniprot-GOA database (http://www.ebi.ac.uk/GOA/, accessed on 20 May 2021) was used for Gene Ontology (GO) annotation. Proteins were classified into three categories according to the GO annotation—biological processes, molecular functions, and cellular compartments. The pathways of the identified ubiquitinated proteins were annotated using the Kyoto Encyclopedia of Genes and Genomes (KEGG) database (http://www.kegg.jp/kegg/pathway.html, accessed on 15 July 2021), and annotation results were mapped on the KEGG pathway database using KEGG online service tool KEGG Mapper. The enrichment results of GO terms and KEGG pathways were employed using a two-tailed Fisher’s exact test. The *p*-value was used to obtain significant enrichment GO terms and KEGG pathways, and *p* < 0.05 was considered to indicate statistical significance. To analyze the sequence characteristics of ubiquitinated sites, 10 amino acids upstream and downstream of all protein Kub sites were extracted and analyzed using the Motif-X software (http://motif-x.med.harvard.edu/motif-x.html, accessed on 15 June 2021). The following parameters were set: motif searching was performed for 20 occurrences, and the Bonferroni-corrected *p*-value was set to 0.000001. PPI networks were constructed using the STRING database (https://string-db.org/, accessed on 21 June 2021) and visualized using the Cytoscape software (version 3.8.0) [33].

### 2.6. Construction of Recombinant Plasmids

To investigate the expression of the ubiquitinated proteins Ta14-3-3 and TaHSP90 under CWMV infection, we fused the green fluorescent protein (GFP) tag to the C-termini of these proteins. We cloned the full-length CDS sequences of *TaHSP90* and *Ta14-3-3* and ligated them into the pCV-C-GFP vector using an In-fusion Cloning kit (Takara Bio, Kusatsu, Japan). The ligation products were cloned into *E. coli*, and three clones of each transformation with recombinant plasmids pCV-Ta14-3-3-C-GFP and pCV-TaHSP90-C-GFP were sequenced, confirming that they contained the full-length CDS sequences of *TaHSP90* and *Ta14-3-3.* Then, the correct recombinant plasmids were transformed into *Agroacterium tumefaciens* strain GV3101 via electroporation (Bio-Rad Gene Pulser, 0.2 cm cuvettes, 25 microF, >2.5 kV; Hercules, CA, USA). All the primers used to create recombinant plasmids are listed in Supplementary Table S1.

### 2.7. Transient Expression and Virus Inoculation

To investigate the effects of Ta14-3-3 and TaHSP90 expression on CWMV infection, we fused a green fluorescent protein (GFP) tag to the C-termini of these proteins. These proteins were infiltrated individually into CWMV-infected *N. benthamiana* leaves via agroinfiltration 4 days after CWMV inoculation, 35S:GFP served as the negative control. After transient overexpression of each gene in *N. benthamiana* epidermal cells, 100 μM MG132 or DMSO as the control was injected into the treated leaves 36 h after inoculation. Green fluorescence was observed under a confocal fluorescence microscope (Nikon, Tokyo, Japan; A1 + A1R). All inoculated leaves were collected three days post-inoculation for detection of CWMV accumulation and ubiquitination levels of host proteins (Ta14-3-3 and TaHSP90).

### 2.8. RNA Extraction and Quantitative Reverse Transcription-PCR (qRT-PCR) Assays

Total RNA was isolated from the leaves of *N. benthamiana* plants inoculated with CWMV, using TRIzol reagent (Invitrogen, Carlsbad, CA, USA) according to the manufacturer’s instructions. Approximately 1 μg of the purified total RNA was reverse-transcribed into cDNA using the First Stand cDNA Synthesis Kit (Toyobo, Osaka, Japan). The relative expression levels of genes were detected via qRT-PCR using the AceQ RT-qPCR SYBR Green Master Mix Kit (Vazyme, Nanjing, China), and fluorescence signals were collected using a 7900 Real-Time PCR system (Applied Biosystems, Foster City, CA, USA). The reaction solution consisted of 10 μL SYBR Green Master Mix (Vazyme), 2 μL cDNA, 0.5 μL 10 μM forward and reverse primer, and 7 μL ddH2O were added to bring the total volume to 20 mL. The amplification conditions were as follows: 95 °C for 3 min, 40 cycles of 95 °C for 30 s, 60 °C for 30 s, and 72 °C for 30 s. Each treatment comprised three biological and four technical replicates, and the ubiquitin-conjugating enzyme was used as an internal reference in this study. All primers used for qRT-PCR are listed in Supplementary Table S2.

### 2.9. Western Blot Analysis of the Ubiquitinated Proteins

Total proteins were extracted from the leaves of each sample to observe green fluorescence. Fresh leaf tissues (0.1 g) were ground into a powder in liquid nitrogen and then added to 500 μL of immunoprecipitation (IP) buffer (150 mM NaCl, 50 mM Tris-HCl [pH 7.5], 5 mM Na_2_EDTA, 10 mM dithiothreitol, 1% (*v*/*v*) Triton X-100, 2 mM Na_3_VO_4_, 2 mM NaF, 1 mM DTT, and 1% protease inhibitor cocktail). The extracts were transferred to new microcentrifuge tubes and centrifuged at 18,000× *g* and 4 °C for 10 min. The GFP-tagged fusion proteins were immunoprecipitated using GFP-trap agarose beads (Sigma-Aldrich, St. Louis, MO, USA). The 25 μL GFP-trap agarose beads were incubated with 400 μL of the extract at 4 °C for 4 h with gentle shaking. Subsequently, GFP-trap agarose was collected and washed thrice with 50 mM Tris-HCl (pH 7.5), and the bound proteins were eluted using 50 μL of a 50 mM Tris-HCl (pH 7.5) buffer. Two hundred micrograms of each protein sample were boiled in SDS-PAGE buffer at 100 °C for 10 min and centrifuged at 18,000× *g* for 10 min. All the protein samples were resolved on a 10% SDS-PAGE gel and transferred to a nitrocellulose (NC) membrane. The accumulation of CWMV and ubiquitination levels of these proteins were determined via immunoblotting using anti-CWMV-CP, anti-ubiquitin (1:2000, rabbit, Abcam, Inc., Atlanta, GA, USA), and anti-GFP (1:5000, mouse, Abcam) as the primary antibodies and an anti-rabbit or anti-mouse secondary antibody conjugated to HRP (1:10,000, Invitrogen, Carlsbad, CA, USA).

## 3. Results

### 3.1. Large-Scale Profiling of Ubiquitination Sites in Wheat Infected with CWMV

**Figure 1:**
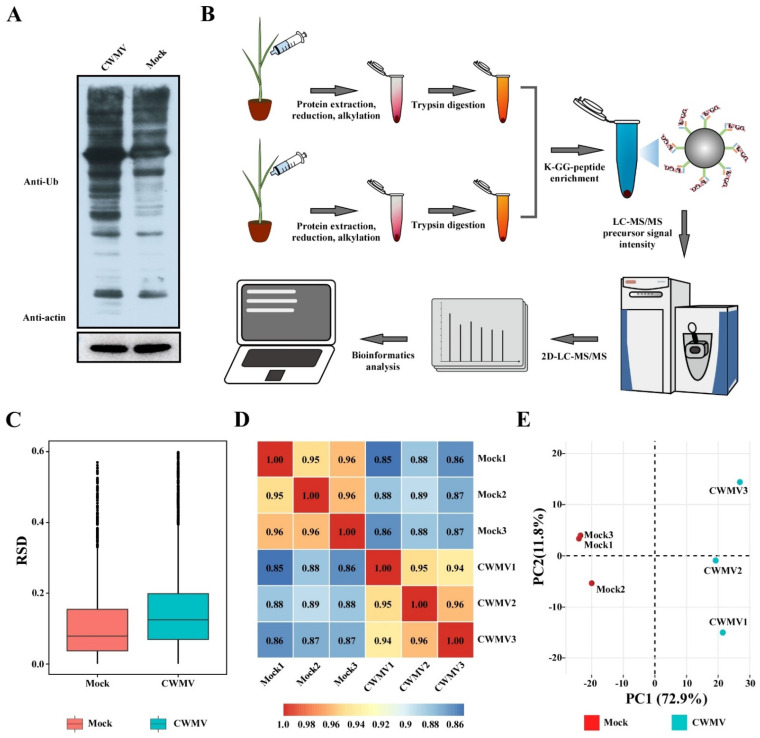
Large-scale identification of lysine-ubiquitinated peptides and proteins in response to CWMV infection in wheat. (**A**) Analysis of the ubiquitination level in CWMV-infected wheat using Western blotting and an anti-ubiquitin antibody; additionally, an anti-actin antibody was used as the loading control. (**B**) Simple workflow for large-scale ubiquitination identification. (**C**) Boxplot shows the relative standard deviation (RSD) of the modified quantitative value among replicate samples. (**D**) The heatmap shows the Pearson correlation coefficient for each sample. Heatmap colors and the number in the figure represent the correlation between two samples. The red or number closer to 1 indicates a better correlation between two samples. Conversely, the blue number closer to 0 indicates a worse correlation between two samples. (**E**) Principal component analysis was performed to evaluate the repeatability and stability of complete LC/MS data for all samples.

To determine whether the overall ubiquitination level in wheat is affected by CWMV infection, we used an anti-ubiquitin-specific antibody and Western blotting to analyze the proteins expressed in CWMV-infected and Mock plants. Compared to that in the Mock plants, the level of protein ubiquitination significantly increased after CWMV infection (Figure 1A). To further identify the specific proteins involved in wheat response to CWMV infection, we performed a proteome-wide analysis of lysine ubiquitination sites and proteins in the CWMV-infected and uninfected samples. An overview of the workflow used in this study is shown in Figure 1B. To confirm the reliability of the LC-MS/MS data, we used the relative standard deviation coefficient (RSD), principal component analysis (PCA), and Pearson’s correlation coefficient to verify measurement repeatability. The RSD and Pearson’s correlation coefficients were close to zero and one, respectively (Figure 1C,D). In addition, PCA revealed a higher degree of clustering between repeated samples in all the samples (Figure 1E). These results indicated that the LC/MS measurements were reliable and could be used for further analyses. In total, 7643 peptides, 2413 proteins, and 4813 ubiquitination sites were identified. Among them, 1255 proteins and 2297 ubiquitination sites were quantified (Table 1).

### 3.2. Analysis of Ubiquitination Sites and Peptide Characteristics

**Figure 2:**
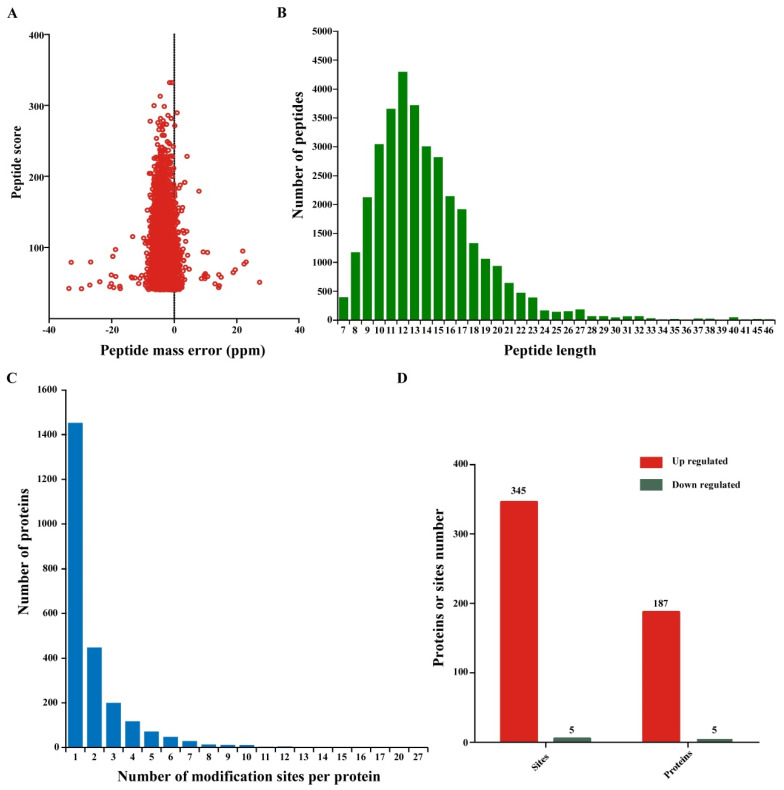
Characteristics of lysine-ubiquitinated sites and proteins in wheat. (**A**) Mass error distribution of all the identified peptides. (**B**) Peptide number and length distribution of all the ubiquitinated peptides. (**C**) Distribution of ubiquitination sites in the ubiquitinated proteins. (**D**) Differential expression of ubiquitinated proteins and site identification in CWMV-infected wheat.

The mass measurement errors for most of the identified peptides were maintained at approximately 10 ppm or less (Figure 2A) to ensure the high measurement accuracy of all the MS data acquired in this study. In addition, we evaluated the number and length of all the identified ubiquitinated peptides. These ubiquitinated peptides had different abundances; over 98.24% of the identified peptide fragments consisted of 7–30 amino acids, with an average length of 14.02 (Figure 2B). Interestingly, we found that ubiquitination sites were not evenly distributed in peptides of different lengths. Approximately 56.5% of the ubiquitinated peptides contained a single ubiquitination modification site (Figure 2C). However, 435, 183, and 172 ubiquitinated proteins contained two, three, and four ubiquitination sites, respectively. Although we identified a large number of ubiquitinated proteins and sites in response to CWMV infection, only 192 ubiquitinated proteins and 350 ubiquitination sites showed significantly increased or decreased expression levels (Log_2_ fold change >1.5 or <−1.5, *p* < 0.05). Specifically, 345 ubiquitination sites in 187 proteins were upregulated, while only five such sites were downregulated (Figure 2D). These results indicated that these differentially expressed ubiquitin-modified proteins may play a positive regulatory role in response to CWMV infection.

### 3.3. Analysis of Ubiquitination Motifs and the Conservation of Ubiquitination Site Characteristics

**Figure 3:**
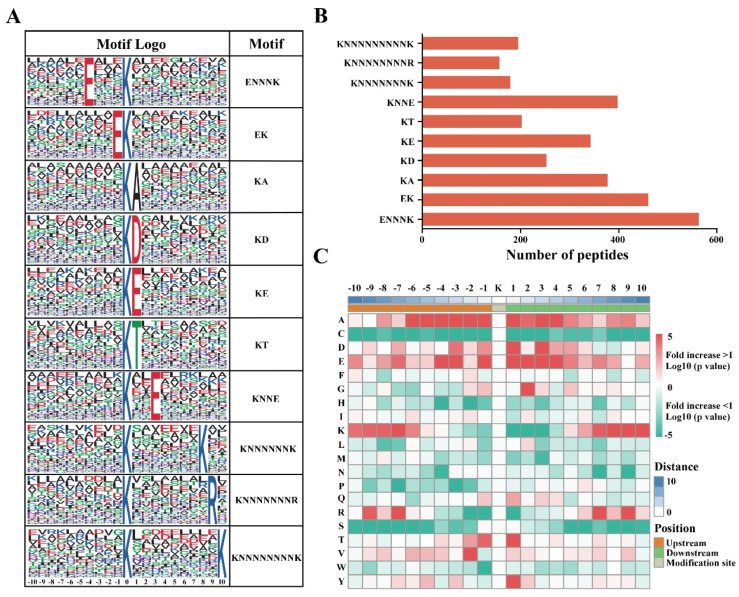
Distribution and characteristics of ubiquitinated lysine motifs and peptides in wheat. (**A**) Identification of ubiquitination motifs and conservative ubiquitination sites in all ubiquitinated peptides. (**B**) Number of ubiquitination motifs on the identified ubiquitinated peptides. (**C**) Amino acid sequences of characteristic ubiquitination sites. The heatmap indicated the expression of significant position-specific amino acids downstream and upstream of the modification sites.

To evaluate whether the sequences of ubiquitinated peptides have a preference after CWMV infection, we used the Motif-X software (http://motif-x.med.harvard.edu/motif-x.html, accessed on 1 June 2021) to search for highly conserved ubiquitination motifs within the identified ubiquitinated peptides. We found that a total of 10 conserved motifs were significantly enriched in the ubiquitinated proteins after CWMV infection (Figure 3A and Table S1), including ENNNKUb, EKUb, KUbA, KUbD, KUbE, KUbT, KUbNNE, KUbNNNNNNK, KUbNNNNNNNR, and KUbNNNNNNNNK, where KUb and N represent modified lysine residues and any amino acid residues, respectively. The results of motif analysis showed that glutamic acid (E), lysine (K), alanine (A), aspartic acid (D), arginine (R), and threonine (T) frequently appeared near the ubiquitin-modified lysine residues under CWMV infection (Figure 3B and Table S1). Interestingly, glutamic acid (E) residues were found near most ubiquitinated lysine residues (K). These results indicated that the proteins with glutamic acid (E) residues around lysine residues (K) were more likely to be modified by ubiquitin. To further analyze the relative abundance of different amino acids surrounding the ubiquitinated lysine sites, we constructed a heatmap for 21-mers (Figure 3C and Table S1). The results showed that alanine (A) and glutamic acid (E) residues were enriched around the modified lysine. Moreover, the residue with the highest frequency at positions −1 and 4 was glutamic acid (E), which was also significantly enriched at positions 1 and 3. These results are consistent with our motif analysis results.

### 3.4. Gene Ontology Pathway Enrichment and Subcellular Localization Analyses of the Differentially Expressed Ubiquitinated Proteins

**Figure 4:**
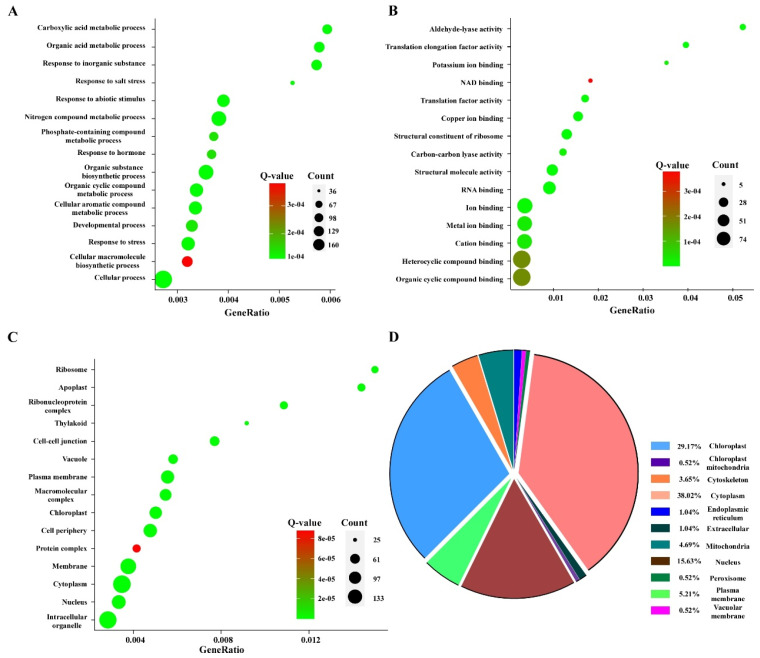
GO enrichment and subcellular localization analyses of the differentially expressed ubiquitinated proteins in CWMV-infected wheat. These ubiquitinated proteins were significantly enriched in biological processes (**A**), molecular functions (**B**), and cellular components (**C**). The Rich factor indicated the ratio of the differentially expressed ubiquitinated proteins to all gene numbers annotated in this pathway term. The Q-value is the corrected *p*-value, ranging from 0 to 1, with lower values suggesting greater intensiveness. The circles represent the number of enriched genes, with the larger circles indicating more enriched genes. (**D**) Subcellular localization of all the identified ubiquitinated proteins.

To better understand the biological functions of the identified ubiquitinated proteins, we used the Gene Ontology (GO) enrichment analysis database to annotate the differentially expressed proteins. These proteins were divided into three categories based on their functions—molecular functions, biological processes, and cellular components. These ubiquitinated proteins were also found to be involved in multiple biosynthetic processes, including metabolic processes, cellular processes, stress responses, biosynthetic processes, and hormone responses (Figure 4A). In the cellular component, most ubiquitin-modified proteins were enriched in the membrane, cytoplasm, nucleus, and chloroplast, which indicated that ubiquitinated proteins play a functional role in these organelles (Figure 4B). The most significant enrichment of molecular functions mainly comprised the binding, cation binding, ribosome structural constituents, and structural molecule activity (Figure 4C). Based on the GO enrichment analysis results, we confirmed the participation of the identified ubiquitinated proteins in a series of biological processes with precise spatial and temporal control. The majority of proteins need to be transported to specific locations to exert their specific functions; thus, we also predicted the subcellular localization of these ubiquitinated proteins. Similar to the results of GO enrichment analysis, 38.02%, 29.17%, 15.63%, 5.21%, and 4.69% of the ubiquitinated proteins were located in the cytoplasm, chloroplast, nucleus, plasma membrane, and mitochondria, respectively (Figure 4D).

### 3.5. Kyoto Encyclopedia of Genes and Genomes (KEGG) Pathway Enrichment and Protein–Protein Interaction (PPI) Network Analyses of Differentially Expressed Ubiquitinated Proteins

**Figure 5:**
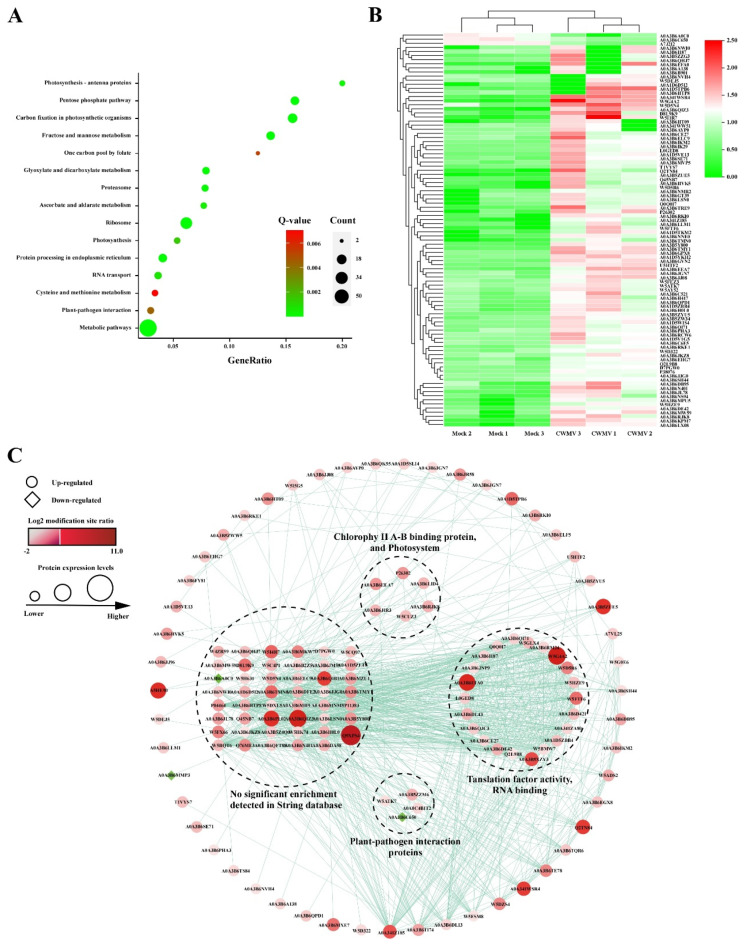
KEGG pathway and interaction network analyses of the differentially expressed ubiquitinated proteins in CWMV-infected wheat. (**A**) KEGG pathway enrichment analysis of the differentially expressed ubiquitinated proteins. The Rich factor indicated the ratio of the differentially expressed ubiquitinated protein numbers annotated in this pathway term to all gene numbers annotated in this pathway term. The Q-value is the corrected *p*-value, ranging from 0 to 1, with lower values suggesting greater intensiveness. The circles represent the number of enriched genes, with the larger circles indicating more enriched genes. (**B**) Heatmap shows hierarchical clustering of significantly differentially expressed genes, which enriched plant–pathogen interaction, response to stress, and response to hormone pathways. The relative protein abundance was normalized by Z-score standardization. (**C**) Interaction networks for all the identified differentially expressed ubiquitinated proteins in CWMV-infected wheat.

Next, we performed a functional enrichment analysis of the identified ubiquitinated proteins using the Kyoto Encyclopedia of Genes and Genomes (KEGG) pathway. As illustrated in Figure 5A, for KEGG analysis, the enriched pathways included metabolic pathways, ribosomes, carbon metabolism, and biosynthesis of secondary metabolites. In addition, the identified ubiquitinated proteins were also found to participate in the plant–pathogen interactions, RNA transport, and other essential cellular processes. These findings suggest that ubiquitination may be involved in plant responses to CWMV infection by regulating essential protein biosynthetic and metabolic pathways. Plant–pathogen interactions, response to stress, and response to hormone pathways are important for the plant immune system to respond to pathogen infections. Therefore, we further analyzed the expression of proteins encoded by the enriched genes involved in these metabolic pathways. As shown in Figure 5B, the majority of ubiquitinated proteins were upregulated upon CWMV infection. These results showed that the genes enriched in these metabolic pathways may play an important role in the response of wheat to CWMV infection.

To further clarify the significance and extent of ubiquitination in response to CWMV infection, a protein–protein interaction network was generated using the Search Tool for Retrieval of Interacting Genes/Proteins (STING) database (https://string-db.org/, accessed on 8 July 2021) and then visualized using the Cytoscape software. A series of sub-networks, including translation factor activity and RNA binding, chlorophyll II A-B binding proteins and photosystems, and stress response-related proteins, were identified using the MCODE tool. The most abundant ubiquitinated proteins in the ubiquitome network are ribosome components, which are widely present in various sub-networks. Moreover, the ubiquitination levels of some ribosomal proteins were increased or decreased after CWMV infection, suggesting that the ubiquitination system can regulate protein translation by increasing or decreasing the abundance of defense response proteins.

### 3.6. Ubiquitylation Patterns and Dynamics of Immunity-Related Proteins under CWMV Infection

**Figure 6:**
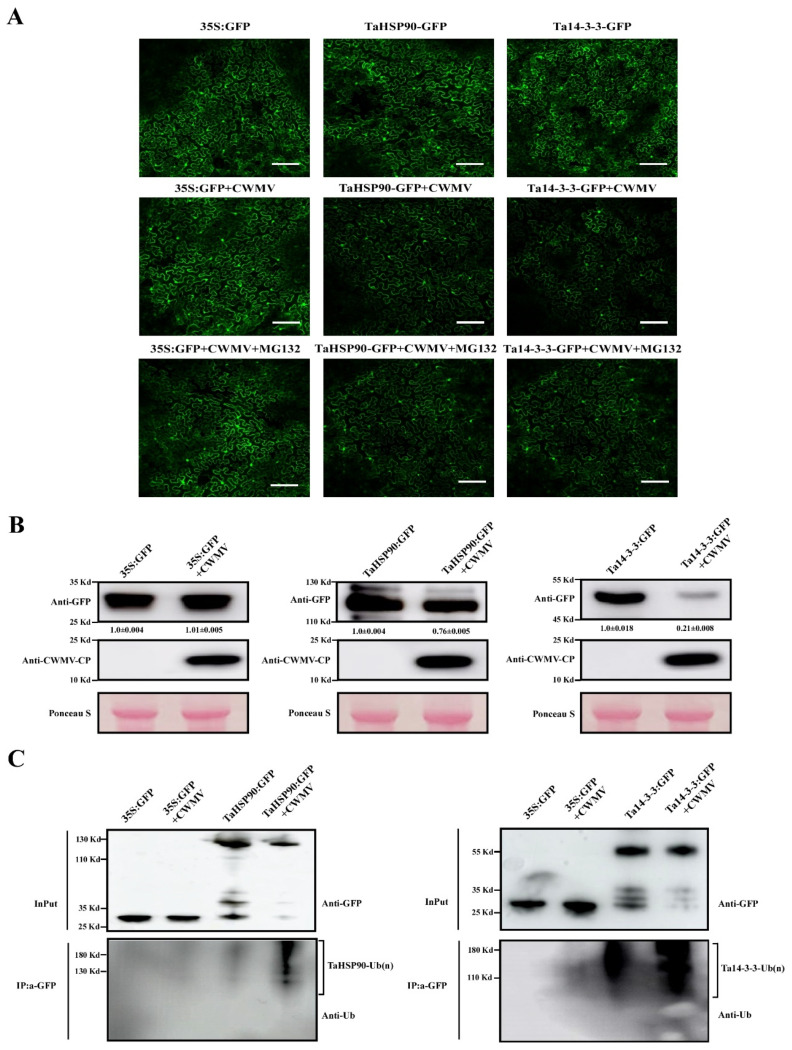
In vivo verification of candidate protein ubiquitination in *Nicotiana benthamiana*. GFP-tagged candidate proteins (including 35S:GFP, Ta14-3-3:GFP, and TaHSP90:GFP) were co-transfected with CWMV in *Nicotiana benthamiana* epidermal cells, where 35S:GFP acts as the negative control. Then, 100 μM MG132 or DMSO as the control was injected into the treated leaves 36 h after inoculation. Subsequently, the fluorescence intensity (**A**) and protein accumulation (**B**) after different treatments were detected using a confocal fluorescence microscope (Nikon, Tokyo, Japan; A1 + A1R) and Western blotting, respectively. The expression levels of CWMV-CP were used to confirm infection in different samples, and then Ponceau S was used to measure protein content in different samples. (**C**) In vivo ubiquitination assay of Ta14-3-3 and TaHSP90 under CWMV infection. GFP-tagged candidate proteins (including 35S:GFP, Ta14-3-3:GFP, and TaHSP90:GFP) were co-transfected in the CWMV-infected *Nicotiana benthamiana* epidermal cells. Protein extracts were immunoprecipitated with GFP-Trap agarose beads (IP: α-GFP), and the ubiquitinated proteins were immunoblotted with anti-ubiquitin antibodies. The input controls are shown in anti-GFP immunoblots.

We further confirmed whether these differentially expressed ubiquitinated proteins are involved in the host response to CWMV infection. The results of GO functional annotation, KEGG pathway enrichment, and protein–protein interaction (PPI) network analyses showed that many proteins that responded to CWMV infection were predicted to interact with Ta14-3-3 and TaHSP90, both of which are related to plant resistance to pathogens. Therefore, we speculated that the Ta14-3-3 and TaHSP90 proteins may be involved in plant defenses against pathogens. We performed ubiquitination verification on two proteins (Ta14-3-3 and TaHPS90) related to pathogen infection during CWMV infection. We successfully cloned *Ta14-3-3* and *TaHPS90* in wheat and fused a green fluorescent protein (GFP) tag at the C-terminus. As shown in Figure 6A, the fluorescence intensity of Ta14-3-3:GFP and TaHSP90:GFP was significantly lower after CWMV infection than control. Surprisingly, the fluorescence intensity of Ta14-3-3:GFP and TaHSP90:GFP did not significantly alter compared to control when treated with protease inhibitor MG132. Similar results were obtained with Western blot analysis. The accumulated levels of TaHSP90 and Ta14-3-3 were reduced under CWMV infection (Figure 6B). To better analyze the ubiquitination levels of TaHSP90 and Ta14-3-3 after CWMV infection, we expressed TaHSP90:GFP and Ta14-3-3:GFP in *Nicotiana* *benthamiana* leaves and immunoprecipitated these proteins using GFP-Trap beads (Figure S1). We then analyzed the levels of ubiquitinated proteins using anti-GFP and anti-ubiquitin antibodies. As shown in Figure 6C, we found that TaHSP90 and Ta14-3-3 ubiquitination levels were markedly increased after CWMV infection compared to those in the control. Taken together, these results show that CWMV infection can significantly enhance the ubiquitination levels of TaHSP90 and Ta14-3-3.

### 3.7. Ta14-3-3 and TaHSP90 Negatively Regulate CWMV Infection in Nicotiana benthamiana

**Figure 7:**
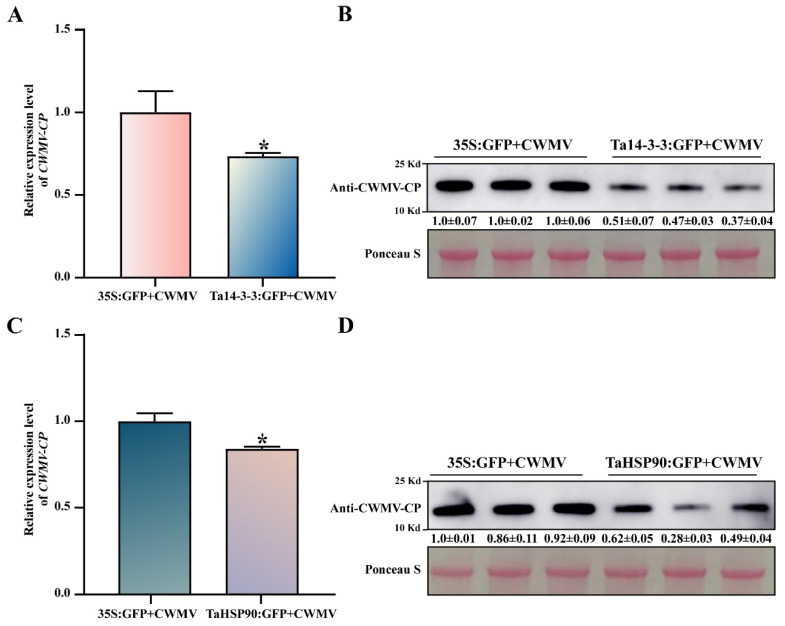
Effects of Ta14-3-3 and TaHSP90 expression on CWMV infection in *Nicotiana benthamiana*. Accumulation of CWMV CP was detected in the Ta14-3-3 and TaHSP90 transient overexpression material through qRT-PCR (**A**,**C**) and Western blotting (**B**,**D**), and 35S:GFP acted as the negative control. * indicates a significant difference between the two treatments (*p* <0.05). Each treatment was repeated thrice, and Ponceau S was used to measure protein content in different samples.

To further explore the role of Ta14-3-3 and TaHSP90 in the host response to CWMV infection, we transiently overexpressed Ta14-3-3 and TaHSP90 in *Nicotiana benthamiana* leaves inoculated with CWMV and detected the accumulation of CWMV at transcriptional and protein levels after 72 h. Compared with that in the control, the accumulation of CWMV was significantly reduced, at both the transcriptional and protein levels, in *Nicotiana benthamiana* plants that transiently overexpressed Ta14-3-3 (Figure 7A,B). Similarly, we found that overexpression of TaHSP90 distinctly reduced the expression and protein accumulation levels of CWMV (Figure 7C,D). These results suggest that Ta14-3-3 and TaHSP90 can act as negative regulators of the response to CWMV infection.

## 4. Discussion

Various abiotic stresses, especially pathogens, threaten the yield, growth, and development of plants [30]. Multiple studies have indicated that ubiquitination, which is one of the most prevalent PTMs, plays an important role in plant response to pathogen infection; however, its regulatory mechanism is still unclear [4,30]. In this study, comprehensive ubiquitome profiling was performed on wheat infected with CWMV. A total of 2297 lysine ubiquitination sites in 1255 proteins were identified. These modified proteins are involved in various metabolic and biological processes. Similar research has also been performed in rice, *Arabidopsis thaliana*, peach, and other eukaryotes [17,34,35,36]. Chen et al. identified 265 ubiquitination sites in 329 proteins under chitin treatment and 269 ubiquitination sites in 343 proteins under flg22 treatment [20]. In addition, Zhang et al. identified 3305 ubiquitination sites in 1562 proteins that respond to heat stress in *Saccharina japonica* [19]. These results suggest that ubiquitination is likely a vital regulatory mechanism for plant responses to pathogens or other stresses. The expression of immunity-related genes can inhibit the normal growth and development of plants [37,38]. During the course of plant–pathogen arms race, pathogens have evolved multiple mechanisms to evade the host immune system. Degradation of immunity-related proteins via hijacking of the host UPS is one such crucial process of pathogen infection [7,31,39]. In this study, we identified 350 differentially expressed lysine ubiquitination sites and 192 differentially expressed ubiquitinated proteins. KEGG and GO enrichment analyses revealed that most differentially expressed genes were responsible immunity-related pathways. This also led to increased ubiquitination of immunity-related proteins during CWMV infection.

Ribosomes are vital organelles involved in protein synthesis in all organisms. Previous research indicates that multiple ribosomal subunits are highly ubiquitinated during biotic and abiotic stress in plants, including *Arabidopsis*, peach, and rice, as well as in humans [17,18]. Kraft et al. examined the effect of nutritional starvation on *Saccharomyces cerevisiae* and found that the ubiquitination of mature ribosomes was selectively degraded by autophagy during starvation [40]. Zhang et al. reported similar results; at least 27 complex-related proteins of the 40S and 60S ribosomes were ubiquitinated during heat stress in *S. japonica* [19]. It has been suggested that the ubiquitinated ribosomal proteins might be an important mechanism for regulating *S. japonica* response to heat stress. In this study, we identified a vast array of ribosome-related proteins that are ubiquitinated after CWMV infection. These observations indicate that the host’s ribosomes likely undergo physical alterations, including improper folding, along with stalling, colliding, and loss of function. Thus, CWMV infection may trigger a series of regulatory mechanisms, including selective degradation by the UPS or autophagy, to maintain protein stability.

Protein phosphorylation is an important post-translational modification that affects protein activity, localization, and stability [41]. In the present study, we found that the ubiquitination level of Ta14-3-3 increased during CWMV infection. Similar results suggest that the RING E3 ligase OsATL 38 negatively regulates the cold stress response in rice via ubiquitination of the OsGF14d (14-3-3) protein [42]. 14-3-3 proteins are involved in a wide range of pathways by serving as scaffold or chaperone proteins. The 14-3-3 proteins play a crucial role in disease resistance and plant immunity [43,44,45]. These studies show that the modification of 14-3-3 protein ubiquitination is an important mechanism for coping with environmental factors. Heat shock proteins (HSP) belong to a general group of molecular chaperones in plants and animals and are involved in a wide range of biological processes, including plant growth and development, immune responses, and other metabolic processes. HSP may also be particularly important in response to pathogen infection [46,47,48,49]. The complex composed of Hsp90, SGT1, and RAR1 enhances disease resistance to cassava bacterial blight (CBB) via precise modulation of autophagy signaling [48]. In addition, a recent study suggests that Hsp90 interacts with SRS1 and WRKY20 to activate salicylic acid (SA) biosynthesis while inhibiting auxin biosynthesis, thereby enhancing disease resistance to CBB in cassava [49]. In this study, we found that TaHSP90, which is crucial for the protein–protein interaction network of differentially ubiquitinated proteins, responded to CWMV infection. Furthermore, the ubiquitination levels of TaHSP90 were significantly increased after CWMV infection. In light of these results, we speculate that TaHSP90 plays a key role in the plant immune system and that the virus hijacks the host UPS system to disrupt an essential component of the innate immune system to complete the infection process.

## 5. Conclusions

Accumulating evidence suggests that PTM plays a crucial role in biological processes such as plant and animal responses to biotic and abiotic stresses and cell signaling. Ubiquitination is a common PTM that is essential for growth, development, and immunity in eukaryotes. However, to date, there have been very few instances of large-scale profiling of the pathogen (especially virus)-induced modification of ubiquitome in wheat. In this study, we identified the ubiquitinated proteins involved in CWMV infection using a combination of affinity enrichment and LC-MS/MS. A total of 1255 ubiquitinated proteins, including 2297 lysine ubiquitination sites, were identified in wheat plants infected with CWMV. However, only 345 upregulated lysine ubiquitination sites in 187 upregulated ubiquitinated proteins and five downregulated lysine ubiquitination sites in five downregulated ubiquitinated proteins displayed significant differential expression after CWMV infection. These differentially expressed proteins were further enriched in response to stress and hormones, plant–pathogen interactions, and other metabolic processes. Interestingly, the ubiquitination levels of Ta14-3-3 and TaHSP90, the key components of the innate immune system, were strikingly enhanced after CWMV infection. This indicates that CWMV disrupts the host’s innate immune system to promote its own replication by hijacking the UPS system.

## Figures and Tables

**Table 1:** Summary of mass spectrum data results.

Total Spectrum	Matched Spectrum	Peptides	Modified Peptides	Identified Proteins	Quantifiable Proteins	Identified Sites	Quantifiable Sites
193,615	30,518	7643	4684	2413	1255	4813	2297

## Data Availability

Not applicable.

## Supplementary Materials

The following supporting information can be downloaded at: https://www.mdpi.com/article/10.3390/v14081789/s1, Table S1: Identification of ubiquitination motifs and ubiquitination sites; Table S2: All primers used in this study; Figure S1: IP products of Ta14-3-3 and TaHSP90 were detected by Western blot.
