# Supplementary material for: Central Role of Ubiquitination in Wheat Response to CWMV Infection
Source: Viruses. 2022 Aug 16;14(8):1789. doi: 10.3390/v14081789 (PMC9412516; doi:10.3390/v14081789)
Supplement: Supplementary file 1 [file viruses-14-01789-s001.zip › Supplementary Figure S1. IP products of Ta14-3-3 and TaHSP90 were detected by Western blot.pdf]

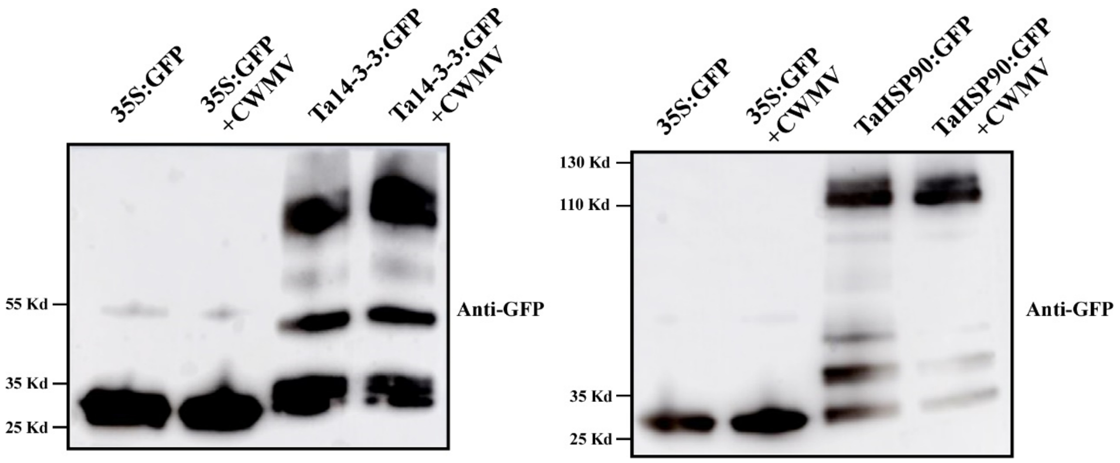

Supplementary Figure S1. IP products of Ta14-3-3 and TaHSP90 were detected by Western blot. 35S:GFP, Ta14-3-3:GFP and TaHSP90:GFP were co-injected into tobacco cells with CWMV, respectively. Proteins extracts were immunoprecipitated with GFP-Trap agarose beads (IP: α-GFP) and IP products were immunoblotted with anti-GFP antibodies.
